# Avatar-based versus conventional patient monitoring with distant vision: a computer-based simulation study

**DOI:** 10.1007/s10877-024-01239-x

**Published:** 2024-11-15

**Authors:** Petar Milovanovic, Julia Braun, Cynthia Alexandra Hunn, Justyna Lunkiewicz, David Werner Tscholl, Greta Gasciauskaite

**Affiliations:** 1 Institute of Anesthesiology, University and University Hospital Zurich, Raemistrasse 100, Zurich, 8091 Switzerland; 2 Epidemiology, Biostatistics and Prevention Institute, University of Zurich, Zurich, Switzerland; 3 Clinic Hirslanden, Institute of Anesthesia and Intensive Care, Zurich, Switzerland

**Keywords:** Avatar-based monitoring, Distant vision, Patient monitoring, Situation awareness, Philips visual patient avatar

## Abstract

Patient monitoring in the perioperative setting can be challenging, especially when monitoring multiple patients simultaneously or managing dynamic situations that require movement around the operating room. We aimed to evaluate whether avatar-based patient monitoring, which presents vital signs in the form of changing colors, shapes and motion, improves remote vital sign recognition compared to conventional monitoring. We conducted a prospective, single-center, computer-based simulation study to evaluate how anesthesia providers recognize vital signs when using the Philips Visual Patient Avatar at different viewing distances (8 and 16 m) compared to conventional monitoring. The primary outcome was the total number of correctly identified vital signs which were compared for the two distances and the two devices using mixed Poisson regression. We analyzed data from 28 anesthesia providers who participated in 112 simulations. The correct recognition rate using the Visual Patient Avatar compared to conventional monitoring at 8 m was increased by 74% (rate ratio 1.74, 95% CI, 1.42 to 2.14, *p* < 0.001) and by 51% at 16-meter viewing distance (rate ratio 1.51, 95% CI, 1.23 to 1.87, *p* < 0.001). We observed scenario-specific superior performance for six vital signs at 8 m. The results provide empirical evidence that avatar-based monitoring can significantly improve the perception of vital signs when using distant vision.

**Supplementary Information:**

The online version contains supplementary material available at 10.1007/s10877-024-01239-x.

## Introduction

Patient monitoring uses electronic devices to collect and display vital signs [1]. This information allows for timely intervention in dynamic situations and has become integral to perioperative [2], emergency, and intensive care medicine [3, 4]. The World Health Organization considers patient monitoring in the presence of a trained anesthesia provider to be essential to the safety of surgical care [5]. Anesthesia providers repeatedly assess the patient, the patient monitoring system, and the surgical field. By integrating these sensory inputs, they develop a mental model of the current situation and its projection into the near future. This cognitive process creates situation awareness [6, 7], which includes the ability to perceive environmental elements, recognize their meaning, and project their future state [8]. Studies have shown that lack of situation awareness causes more than two-thirds of anesthesia-related complications, with perceptual errors being the most common subtype [9, 10].

The current design of patient monitoring systems does not optimally support the care providers’ perception of impending critical events [11]. Conventional monitoring relies on a single-sensor-single-indicator principle, a technology-oriented form of information presentation in which individual parameters are measured and displayed as discrete numbers and waves [12, 13]. Care providers must process, integrate, and interpret each vital sign separately before they can decipher its meaning [14]. Moreover, the numerical range of some vital sign values overlaps (e.g., the number 95 may correlate with a physiologic heart rate, oxygen saturation or systolic blood pressure value) [15]. The spatial and color layout of vital signs is adjustable, which can be an additional distraction when reading their values sequentially [16]. On the other hand, customization of vital sign layouts allows for adaptation to individual or local preferences, which can enhance the intuitiveness and interoperability of patient monitoring systems [17]. All of this, combined with the limited capacity of short-term memory, can make it difficult to respond promptly to changes in vital signs and can delay potential treatment [18, 19].

**Fig. 1:**
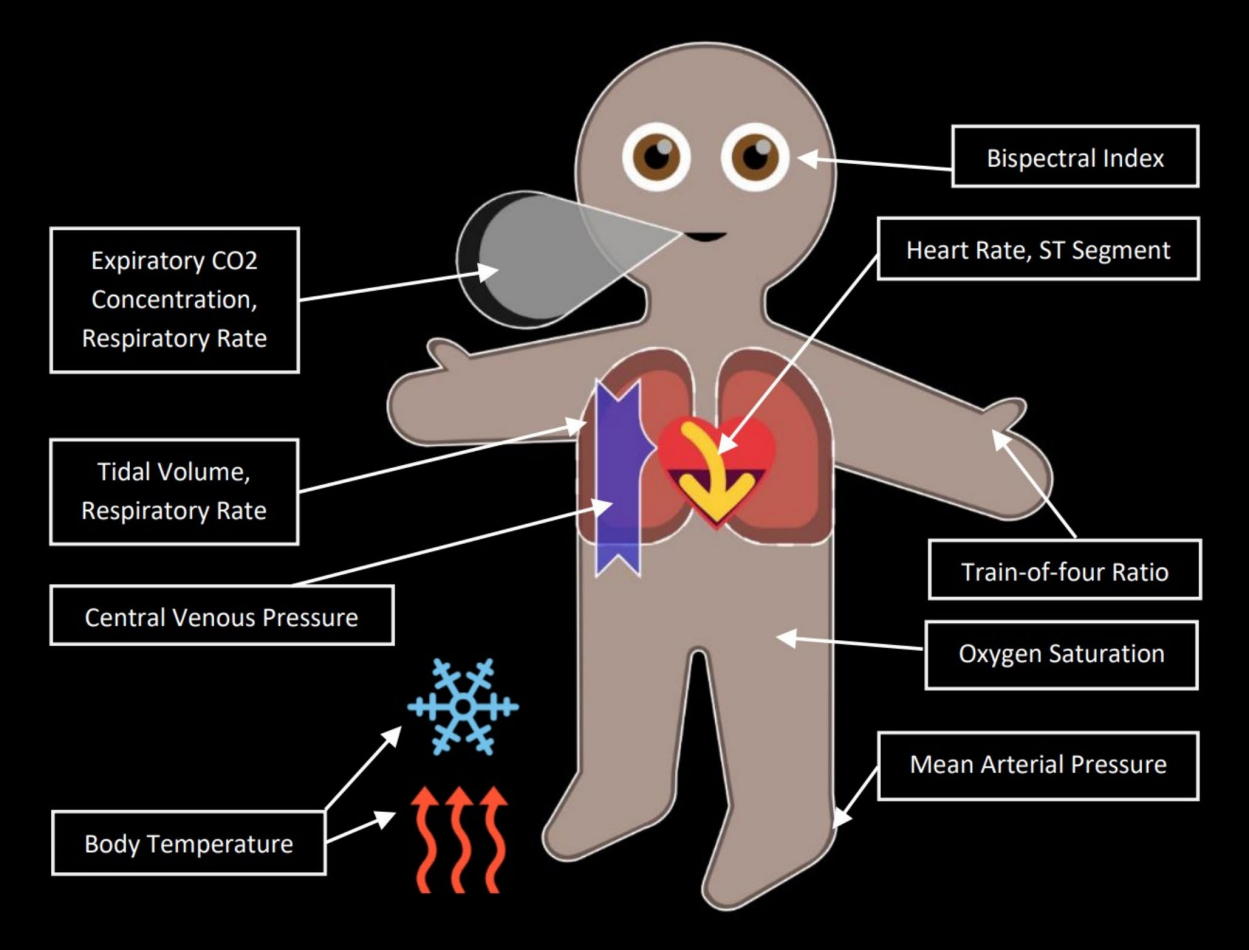
Philips Visual Patient Avatar. Vital signs and their respective visualizations

The growing recognition of the limitations of conventional monitoring systems led to the developments resulting in the Philips Visual Patient Avatar [20]. This technology integrates principles of logic, cognitive engineering for situation awareness, information architecture and user-centered interface design [21, 22]. The Visual Patient Avatar transforms numbers and waveforms into visual objects and animations (Fig. 1). In addition to directly displaying information, i.e., showing the information in a way that corresponds to the phenomena expected in the real patient, it categorizes vital sign data into safe and unsafe (and further into low or high). It also offers simultaneous visualizations for a single vital sign (e.g., respiratory rate corresponds to the speed of the animation of the lungs breathing and the avatar exhaling a cloud of carbon dioxide). For a detailed description of the Philips Visual Patient Avatar please refer to the Online Resource 2.

Several computer-based and high-fidelity simulation studies showed that the Visual Patient Avatar improves vital sign recognition, diagnostic confidence and reduces perceived workload compared to conventional monitoring [23–25]. An eye-tracking study confirmed these findings for peripheral vision. The authors attributed this success to the vivid animations that facilitate vital sign recognition with peripheral vision [15]. We suggest a new concept - patient monitoring with distant vision. The purpose of this study was to test the hypothesis that the Visual Patient Avatar improves vital sign recognition with distant vision compared to conventional patient monitoring technology.

## Methods

### Approval and consent

The Cantonal Ethics Committee of Zurich in Switzerland reviewed the study protocol and issued a declaration of non-jurisdiction, specifying that the research project does not fall within the scope of the Human Research Act (Business Management System for Ethics Committees Req-2023-00580). All participants signed written informed consent to use their data for research purposes. Participation in the study was voluntary and without financial compensation.

### Study design

We conducted a researcher-initiated, prospective, single-center, computer-based, within-subject simulation study. We aimed to investigate how anesthesia providers recognize vital signs using distant vision (viewing distances of 8 and 16 m) with the Visual Patient Avatar compared to conventional monitoring. Data collection occurred over three consecutive weeks in May and June 2023 at the Institute of Anesthesiology, University Hospital Zurich, Switzerland. The study center is the first institution where the Visual Patient Avatar has been implemented in clinical practice (March 2023). During the initial phase of the technology implementation, lectures were held to explain the concept of the technology and training sessions were organized to provide information on its use. An educational video and a short user guide were made available on the study center’s intranet (Online Resources 1 and 2).

### Data collection and simulation settings

We initially derived two sets of scenarios (i.e., Scenarios 1 and 2) of eleven vital sign values reflecting two possible patient states under general anesthesia. We deliberately chose vital sign values that were either safe or unsafe regarding their respective thresholds. We then randomly assigned these values to each scenario to avoid pattern recognition (i.e., blind estimation based on correlation with other vital sign values). The vital sign sets for both scenarios are listed in Online Resource 3.

**Fig. 2:**
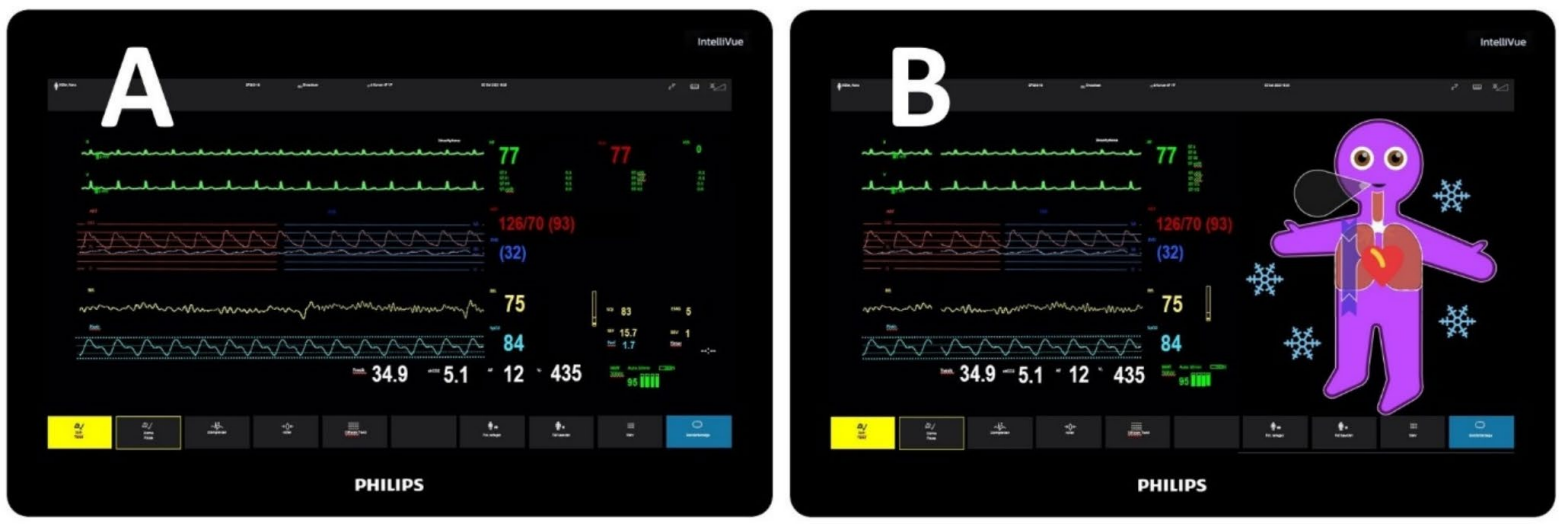
Display modalities. Screenshots taken of the simulation videos for scenario 1, including the conventional modality (**A**) and the split-screen modality (**B**)

We incorporated the scenarios into the respective simulation videos using two display modalities: a conventional modality, in which we presented only the conventional patient monitoring, and a split-screen modality, in which we presented both technologies (conventional monitoring and Visual Patient Avatar) simultaneously side by side, as shown in Fig. 2.

**Fig. 3:**
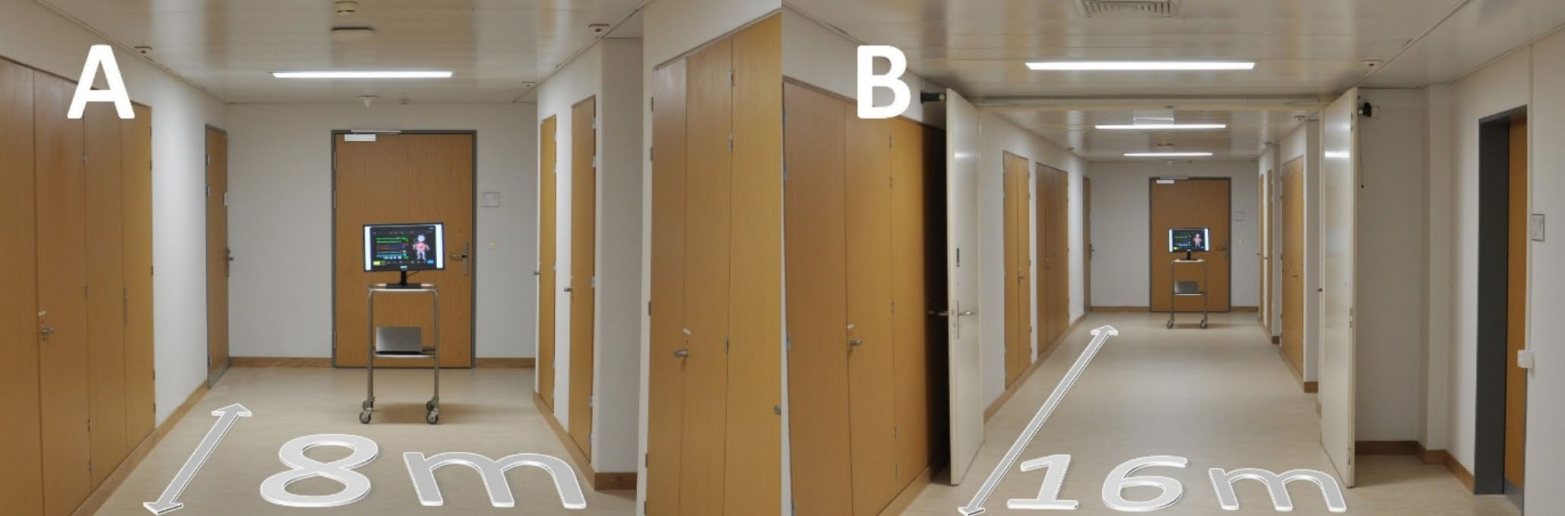
Study setup. Photographs taken from the participants’ perspective, standing at 8- (**A**) and 16- meter (**B**) viewing distance from the computer monitor used to display the simulation videos. The superimposed gray arrows correspond to the viewing distance. Abbreviations: 8 m: 8-meter viewing distance; 16 m: 16-meter viewing distance

Before the simulation session began, participants completed a non-identifying survey that included questions about age, gender, eyesight, role (nurse, resident, consultant), and years of work experience in anesthesia. The sessions were performed in a quiet hallway, free of distraction from the clinical areas of the hospital. We used a measuring tape to determine the participant’s position according to the viewing distance (8 and 16 m). Figures 2 and 3 illustrates the study setup.

During each simulation video playback we asked the participants to simultaneously recognize vital sign values. The available nominal scale level measurement options for seven vital sign values (e.g., pulse rate, central venous pressure) were ‘too low,’ ‘safe,’ ‘too high,’ or ‘no recognition.’ For others (e.g., oxygen saturation, brain activity), the options were either ‘safe,’ ‘unsafe,’ or ‘no recognition.’ We limited the duration of each simulation video to two minutes, after which it ended and the participant had to recall the remaining values without seeing the videos. Only a few participants were unable to complete the task within the given time. Although this task may appear to be partly a test of short-term memory, in this case it is simply an unavoidable part of the simulation study when completing the questionnaire.

**Fig. 4:**
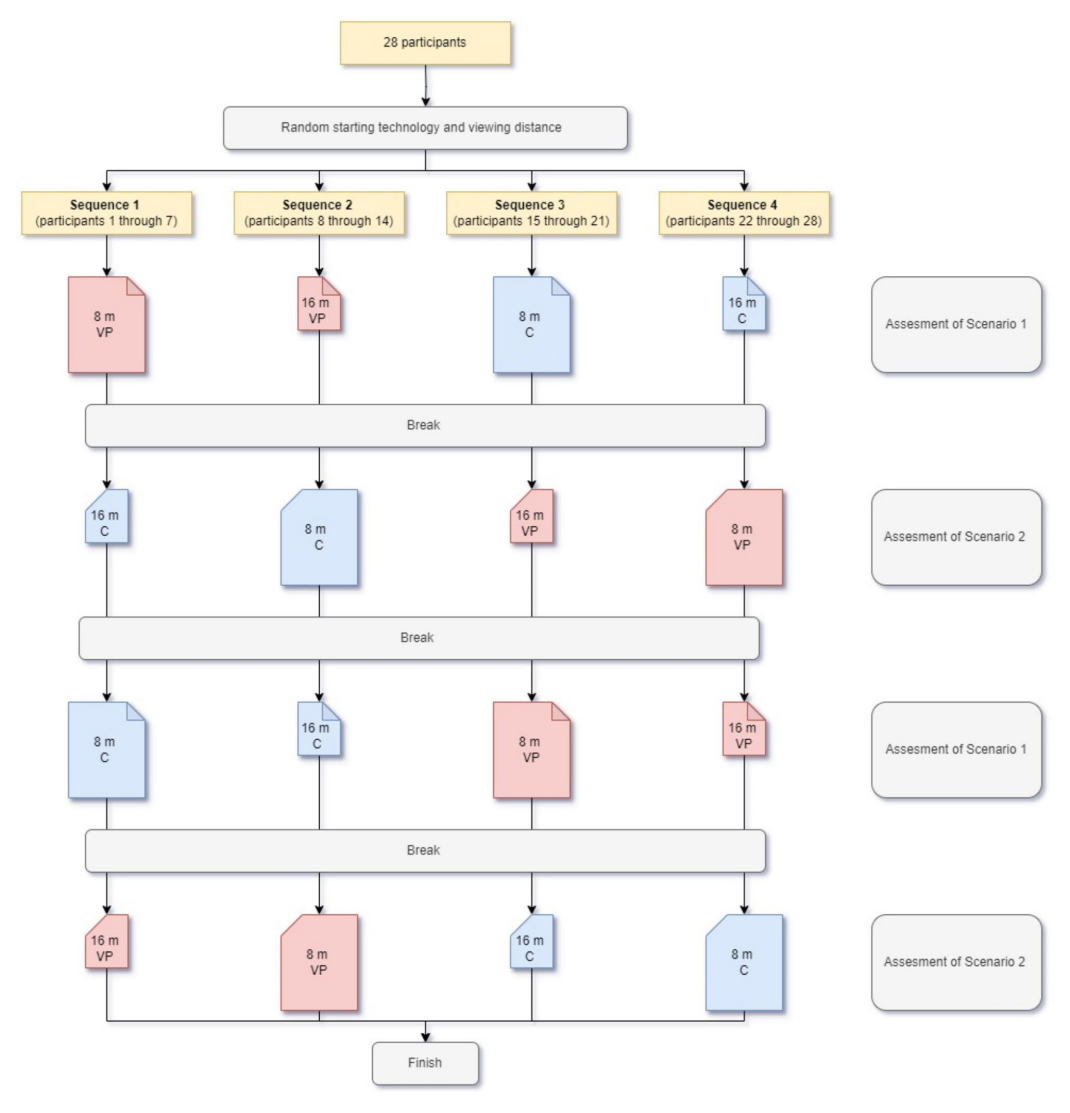
Simulation session sequences flowchart. Each participant evaluated four simulation videos sequentially, thereby alternating the scenario and viewing distance at every turn. By changing the starting technology and viewing distance we created four different simulation session sequences (can be read from top to bottom in the flowchart) and divided the participants accordingly. Each simulation video in the flowchart is described with its corresponding scenario (on the right), technology (red for Philips Visual Patient Avatar and blue for conventional monitoring), and viewing distance (larger pictogram size for 8-meter viewing distance and smaller pictogram size for 16-meter viewing distance). Abbreviations: 8 m: 8-meter viewing distance; 16 m: 16-meter viewing distance; C: conventional monitoring; VPA: Philips Visual Patient Avatar

Each scenario appeared twice, once for each monitoring technology. To keep participants blind to this fact, we arranged the resulting four simulation videos sequentially so that the scenarios alternated. We additionally alternated the viewing distance between each video, thereby creating four distinct simulation session sequences. Therefore each technology-viewing distance pairing (e.g. Philips Visual Patient Avatar at 8 m) for a given scenario (e.g. Scenario 1) was evaluated by a total of 14 participants (e.g. 1 through 7 and 15 through 21). Figure 4 provides a flowchart detailing this procedure.

### Outcomes

We defined the sum of correctly recognized vital signs as the primary outcome. A secondary outcome was the binary variable “correct identification” for each vital parameter and situation.

### Experimental setup and equipment configuration

We used Microsoft PowerPoint (Microsoft Corporation, Redmond, WA, USA) to create digital mockup templates for each technology. The layout of the vital signs closely resembled the Philips IntelliVue MX750 patient monitor (Koninklijke Philips NV, Amsterdam, The Netherlands) routinely used in our study center. We generated the Visual Patient Avatar animations using an internally developed simulator software.

All simulation videos were performed on a Swift3 14-inch laptop computer (ACER, Inc. Taipei, Taiwan). To emulate the size of the Philips Intellivue MX750 monitor, we mirrored the laptop screen onto an external Samsung S24E450 computer monitor (Samsung Electronics Co., Ltd., Seoul, South Korea) in full high resolution (1920 × 1080 pixels) at 60 frames per second, without audio.

Participants assessed vital signs simultaneously with each video using the iSurvey questionnaire application (Harvest Your Data, Wellington, New Zealand) on an iPad- (Apple Inc., Cupertino, CA, USA).

### Data analysis

For descriptive statistics, we provide means and standard deviations, medians with interquartile ranges for continuous data, and numbers with percentages for categorical data.

We used a mixed Poisson regression model to compare the difference in correctly recognized vital signs depending on the categorical variables of monitoring technology and viewing distance. The inclusion of an interaction term allowed us to assess the effect of each combination of variables (Visual Patient Avatar from an 8 m viewing distance, conventional monitoring from a 16 m viewing distance and Visual Patient Avatar from a 16 m viewing distance) relative to the reference category (defined as conventional monitoring from an 8 m viewing distance). Notably, this model accounted for repeated measurements obtained from the same participant by including a random intercept per participant. Furthermore, we implemented a second model adjusted for the respective scenario to examine the influence of scenarios as an additional variable on the outcome.

We conducted a pilot study before recruiting participants. To determine the required sample size, we took the resulting rate ratio of 1.35 to calculate 1000 simulated data sets. A sample size of 20 participants per data set could demonstrate a statistical power of over 90%.

We used Microsoft Excel spreadsheets (Microsoft Corporation, Redmond, WA, USA), Prism 9 (GraphPad Software Inc., San Diego, CA, USA) and R version 4.0.5 (R Foundation for Statistical Computing, Vienna, Austria) to manage and analyze our data. We generated the participant flowchart using draw.io (JGraph Ltd., Northampton, UK). We used a Nikon D90 D-SLR camera (Nikon Corporation, Minato, Tokyo, Japan) to acquire photographic material, which we then edited using GIMP (GNU Image Manipulation Program, GNU General Public License).

## Results

### Study and participant characteristics

We enrolled 30 anesthesia providers who completed 120 simulations. However, due to technical issues with the data storage software, questionnaire data from two participants were not available for analysis. Consequently, we analyzed data from 28 participants, representing 112 simulations. Table 1 shows the characteristics of the participants.

**Table 1:** Participant characteristics

Participants included in data analysis, *n*	28
Male, n (%)	17 (60.7)
Female, n (%)	11 (39.3)
Age of participants (years), median (IQR)	33 (29–35)
Work experience (years), median (IQR)	4 (1.5-7.0)
Professional group	
Interns, n (%)	3 (10.7)
Nurse anesthetists, n (%)	9 (32.2)
Resident physicians, n (%)	13 (46.4)
Consultant physicians, n (%)	3 (10.7)
Self-reported eyesight	
Normal, n (%)	17 (60.7)
Near-sighted, n (%)	10 (35.7)
Far-sighted, n (%)	1 (3.6)

IQR: interquartile range

### Primary outcome

**Fig. 5:**
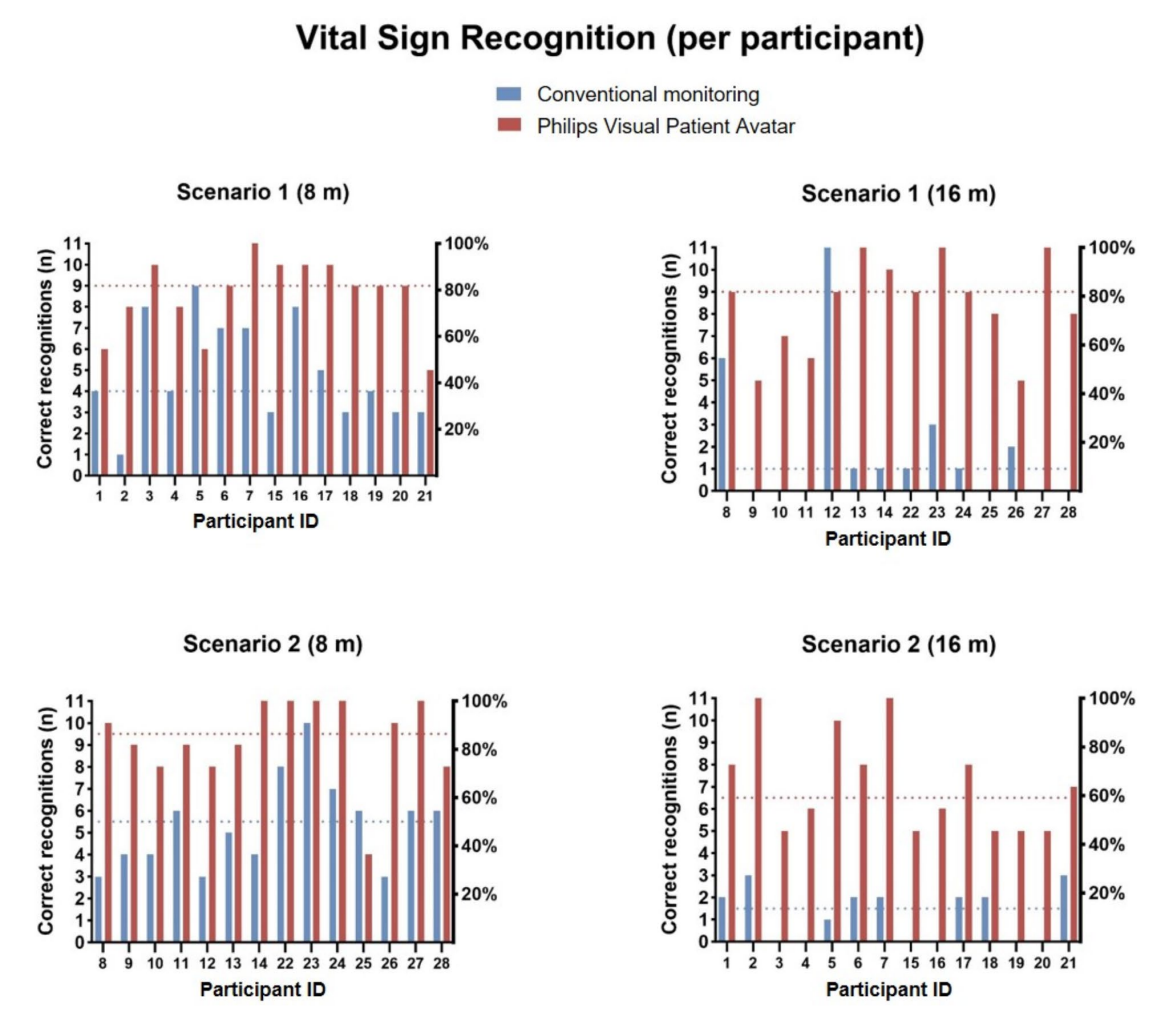
Vital sign recognition per participant. Grouped column chart showing the performance of participants in regard to scenario and viewing distance. Each participant is marked appropriately with a consecutive number (Patient ID 1–28; as shown in Fig. 4). The median number of correct recognitions for each technology is shown with the colored dotted line. Abbreviations: 8 m: 8-meter viewing distance; 16 m: 16-meter viewing distance

Using the Visual Patient Avatar, the number of correctly recognized vital signs was higher from an 8-meter viewing distance. The mixed Poisson model shows a 74% increase in recognized vital signs at 8 m (rate ratio 1.74, 95% CI, 1.42 to 2.14, *p* < 0.001) when using the Visual Patient Avatar compared to conventional monitoring. Figure 5 illustrates the results for each scenario per participant.

Of all 56 descriptive intra-participant comparisons of the performance using the conventional or the Visual Patient Avatar, the participants performed better using the latter in all but three cases. In scenario 1, from an 8-meter viewing distance, the median number of correctly recognized vital signs using the Visual Patient Avatar was five vital signs higher, rising from a median [IQR] of 4 [3, 7] with conventional monitoring to 9 [8, 10] with the Visual Patient Avatar. Similarly, in scenario 2, at a viewing distance of 8 m, it was higher by four vital signs, increasing from 5.5 [4, 6] with conventional monitoring to 9.5 [8.25,11] with the Visual Patient Avatar. The differences between the two technologies were even more prominent when assessed from 16 m. In scenario 1, the median number of correct recognitions with the Visual Patient Avatar was higher by eight vital signs, from 1 [0,1.75] with conventional monitoring to 9 [7.25,9.75] with the Visual Patient Avatar. In scenario 2, it was higher by five vital signs, increasing from 1.5 [0,2] with conventional monitoring to 6.5 [5, 8] with the Visual Patient Avatar. In the mixed Poisson regression model, the correct recognition rate compared to the reference category (conventional monitoring from an 8-meter viewing distance) was 74% higher from an 8-meter viewing distance and 51% higher from a 16-meter viewing distance. Table 2 presents these results. After including the respective scenario as an influential variable in the second model, we obtained consistent results, negating the effect of either scenario on the primary outcome.

**Table 2:** Primary outcome results of the mixed Poisson regression model

	rate ratio	95% confidence interval	*p*-value
VPA + 8 m	1.74	from 1.42 to 2.14	< 0.001
C + 16 m	0.30	from 0.21 to 0.42	< 0.001
VPA + 16 m	1.51	from 1.23 to 1.87	< 0.001

VPA: Visual Patient Avatar; C: conventional monitoring; 8 m: 8-meter viewing distance; 16 m: 16-meter viewing distance; 95% CI: 95% confidence interval

### Secondary outcome

**Fig. 6:**
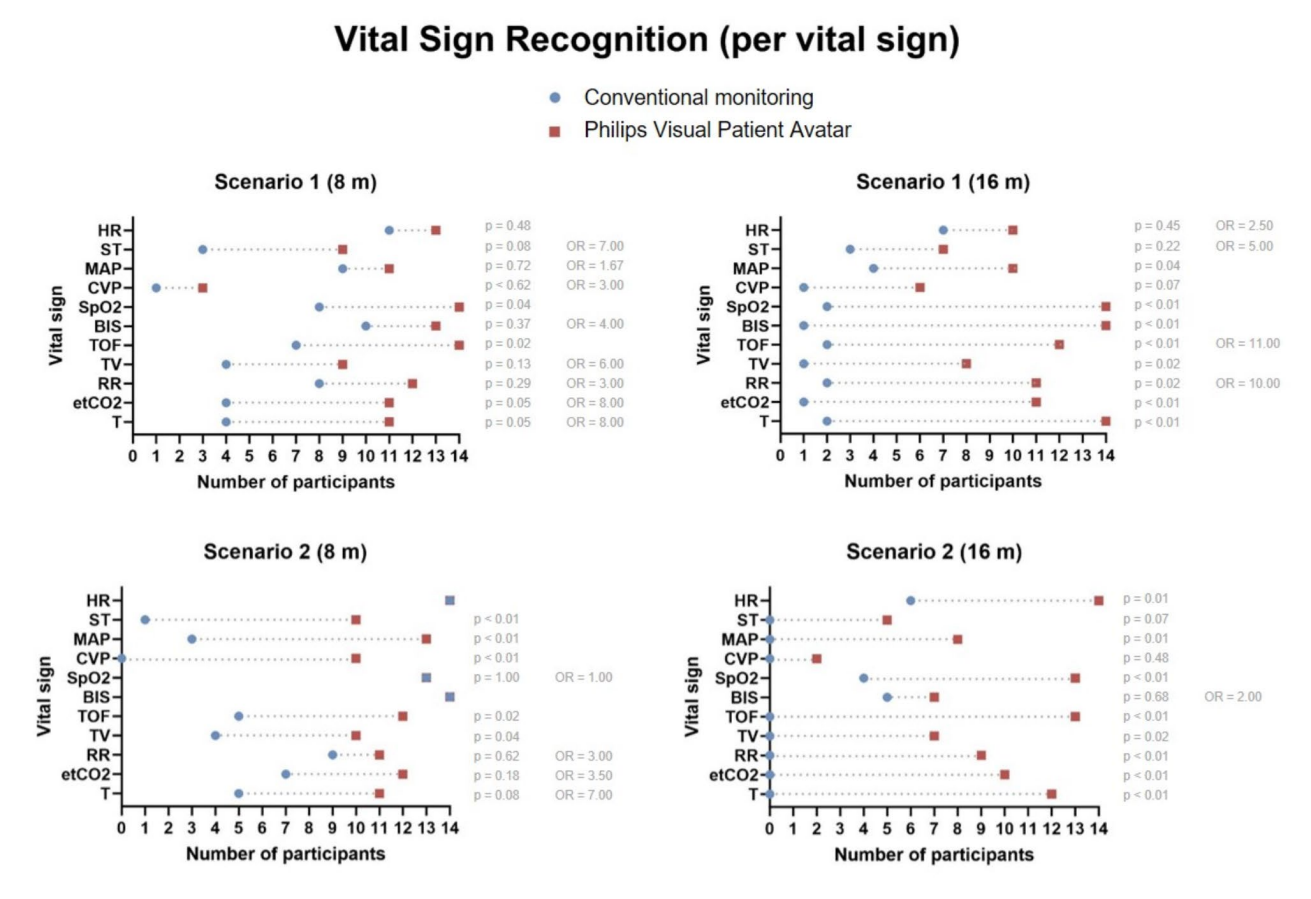
Vital sign recognition per scenario. Abbreviations: HR: heart rate; ST: ST segment; MAP: mean arterial pressure; CVP: central venous pressure; BIS: bispectral index; TOF: train-of-four ratio; SpO2: oxygen saturation; RR: respiratory rate; TV: tidal volume; etCO2: expiratory carbon dioxide concentration; T: body temperature; 8 m: 8-meter viewing distance; 16 m: 16-meter viewing distance; OR: odds ratio; p: p-value

When looking at the different vital signs separately, there was no case where the conventional monitoring showed a better performance than the Visual Patient Avatar. We furthermore provided evidence for better performance of the Visual Patient Avatar when assessing train-of-four ratio in both scenarios and both viewing distances. Figure 6 shows the results for each vital sign per scenario and viewing distance. Dot plot presents the number of participants who correctly identified individual vital signs. The difference in the number of participants using both technologies is represented by the dotted line. Odds ratios are missing where they could not be calculated due to a value of zero in the underlying Table (0 participants who had an incorrect response with Visual Patient Avatar but a correct response with conventional technology). Similarly, we did not include p-values where they could not be calculated because the matrix on which the test was based had only one non-zero entry.

In scenario 1, there was strong evidence for two and moderate evidence for further two vital signs performing significantly better at an 8-meter viewing distance. Scenario 2 also had five vital signs with strong evidence for superior performance, four differed from scenario 1. Apart from the central venous pressure and the ST segment, all vital signs supported improved performance at the 16-meter viewing distance. In both scenarios we saw significantly better results in eight out of the eleven vital signs.

## Discussion

### Principal findings

The current study demonstrated an advantage for the Visual Patient Avatar with distant vision. The validation of these findings in both scenarios ensures the consistent and reliable applicability of the Visual Patient Avatar with distant vision.

Conventional and avatar-based patient monitoring technologies can be understood in neurocognitive terms as a comparison between visual symbols (Arabic numerals) and visual objects (vital sign visualizations of the Visual Patient Avatar) [26]. Numbers are arbitrary symbols that are culturally acquired in early childhood, enabling literate adults to recognize them instantly [27, 28]. In the context of our study, this implies that remote vital sign recognition using conventional monitoring relies solely on visual sharpness, i.e. visual acuity [29]. On the other hand, visual object recognition depends on visual acuity and the visual processing of its shape, color, and motion [30].

Visual acuity is the relationship between the size of a stimulus and its detection [31]. Increasing distance reduces the relative size of the stimulus, leading to a progressive loss of detail. There are three types of visual acuity: minimum visible, minimum resolvable and minimum discriminable [29]. Minimum visible refers to detecting the presence of a visual stimulus. Minimum resolvable visual acuity regards distinguishing details, such as the visual stimulus’s form, shape and pattern (such as in numerals). Minimal discriminable acuity finally relates to detecting a discontinuity of alignment, i.e., the relative location of more than one object [29, 32, 33] (Online Resource 4). Both minimum visible and minimum discriminable visual acuity have lower thresholds than minimum resolvable visual acuity [29]. Therefore, at a given viewing distance, a person is more likely to notice a visual stimulus of a certain size, or the relative shift in position of an object by the same size increment, than to accurately recognize the exact shape of a visual stimulus (such as a letter or numeral) when its details are also of that size. Using these principles, we applied the type of visual acuity to each vital sign visualization of the Visual Patient Avatar (Online Resource 5). Only the train-of-four ratio falls under the minimum resolvable type. Six vital signs are of the minimum visible and four of the minimum discriminable type. The low threshold associated with these visual acuity types validates the better performance of the Visual Patient Avatar within the primary outcome.

To investigate and further differentiate the effect of distance monitoring, we analyzed the number of participants with correct vital sign recognition per individual vital sign. Notably, the vital sign of the minimally resolvable type (train-of-four ratio) is the only one that consistently improves across scenarios and viewing distances. We explain this finding according to the traditional, primarily shape-driven theory of visual object recognition [34]. Recent research, however, extends the role of color from a “useful cue” towards significantly facilitating recognition especially with extensive experience within a specific category domain (e.g., bird watching) [35, 36]. This argument is reflected in the fact that oxygen saturation and body temperature, using bold colors, had significantly better results in three out of four scenario-distance pairings. Motion is the third component of visual object recognition and can be added or subtracted to an object without affecting its shape and color [30]. The individual visualizations of the Visual Patient Avatar do not move, but some perform a frequency-dependent pulsating animation. The speed of the animation is perceived through another, less understood process called visual temporal processing [37].

### Implications and applications

Research indicates that anesthesia providers only directly observe the monitoring device approximately 5% of the time [38, 39]. One of the reasons for this is that anesthesia providers must spend a lot of time on critical tasks that require physical movement around the operating room, for instance, equipment checks, inspecting blood levels in surgical suction canisters and assessing the surgical field from different angles. The patient monitoring technology must support the anesthesia providers in adequately performing patient care by enabling an effective information transfer from the monitoring device to the provider. The current study showed that Visual Patient Avatar allows anesthesia providers to assess the patient`s vital signs without being close to the monitoring device.

Moreover, distant vision monitoring may be essential for supervising multiple patients simultaneously. When going past the operating theatre or resuscitation room, care providers can swiftly evaluate the patient from a distance and assess the need for intervention. This idea can expand to recovery rooms. Such a comprehensive overview enhances patient safety and optimizes healthcare resources, ensuring timely interventions and personalized care delivery.

### Limitations

There are several limitations to this study. First, while computer-based laboratory studies allow for controlled experiments, they may only partially capture the dynamic nature of clinical practice. This simulation did not include audio alarms or color highlighting of unsafe vital signs, features of any state-of-the-art conventional monitoring device. We deliberately chose to exclude these alarm features and focus solely on the visual perception of a given set of vital signs. Given Roche et al. demonstrated that standard state-of-the-art auditory alarms achieved only 20% alarm detection accuracy [40], we suggest that the inclusion of additional current-day standard auditory cues in this study would not result in significantly different findings beyond the 20% maximum theoretical change. Additionally, in environments such as recovery rooms or intensive care units, where multiple heart rate and oxygen saturation sounds may overlap, simultaneous playback could create confusion rather than enhance clarity, limiting the practicality of such cues in these settings.

Moreover, there are no established guidelines or reference materials regarding viewing distances for clinical monitors. To our knowledge, there are no industry or government standards that define the maximum viewing distance for medical monitors, nor did we find any previous studies that address this issue. Anesthesiologists typically work within 1–2 m of the anesthesia workstation, while in our hospital’s recovery room, the central desk is located approximately 15 m from the farthest bed in a 20–30 m room. Therefore, the viewing distances we selected in our study were based on these routine clinical observations rather than experimental data.

Furthermore, although we have demonstrated significant performance improvements in all vital parameters, further research is needed to make these results more consistent and independent of viewing distance and scenario.

## Conclusion

This study introduces the concept of distant vision monitoring. Our findings strongly support that avatar-based patient monitoring technology such as the Philips Visual Patient Avatar can significantly improve remote vital sign recognition. Further research is needed to translate these findings into everyday clinical practice, assess their impact on anesthesia performance, and evaluate their influence on patient outcomes.

## Electronic supplementary material

Below is the link to the electronic supplementary material.

Supplementary Material 1

## Data Availability

The datasets used and/or analyzed during the current study are available from the corresponding author on reasonable request.

## Abbreviations

8m: 8-meter viewing distance
16m: 16-meter viewing distance
C: Conventional monitoring
VPA: Visual Patient Avatar
min^− 1^: per minute
mV: millivolt
mmHg: millimeter mercury
ml: milliliter
kPa: kilopascal
°C: degree Celsius
CI: Confidence Interval
HR: Heart Rate
ST: ST segment
MAP: Mean Arterial Pressure
CVP: Central Venous Pressure
BIS: Bispectral Index
TOF: Train-of-Four ratio
SpO2: Oxygen saturation
RR: Respiratory Rate
TV: Tidal Volume
etCO2: expiratory Carbon Dioxide concentration
T: body temperature
OR: Odds Ratio
IQR: Interquartile Range
