# Supplementary material for: Avatar-based versus conventional patient monitoring with distant vision: a computer-based simulation study
Source: J Clin Monit Comput. 2024 Nov 15;39(5):1065–75. doi: 10.1007/s10877-024-01239-x (PMC12474633; doi:10.1007/s10877-024-01239-x)
Supplement: Supplementary file 1 — Supplementary Material 1 [file 10877_2024_1239_MOESM1_ESM.docx]

# Supplementary Information


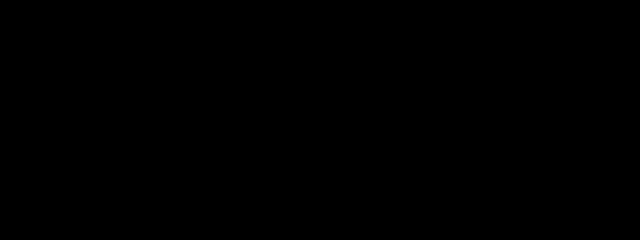


**Online Resource 1** Educational video providing a detailed demonstration of the Philips Visual Patient Avatar and its representation of individual parameters within and outside their normal ranges

May also be retrieved from the following link:

<https://onedrive.live.com/?authkey=%21AO9AhzOXksfxHlE&cid=4CDC70FFF5992E39&id=4CDC70FFF5992E39%21267714&parId=4CDC70FFF5992E39%21274697&o=OneUp>

**Online Resource 2** Short user guide to the Philips Visual Patient Avatar, available on the study centre’s intranet

| Vital sign | Measuring unit | Range | Scenario 1 | Scenario 2 |
| --- | --- | --- | --- | --- |
| HR | min^-1^ | 50-95 | 75 | 110 |
| ST | mV | -0.2-0.2 | 0.0 | 4.5* |
| MAP | mmHg | 65-100 | 93 | 58 |
| CVP | mmHg | 4-12 | 32 | 3 |
| BIS | / | 40-60 | 75 | 45 |
| TOF | % | < 80 | 95 | 0 |
| SpO2 | % | < 94 | 85 | 99 |
| RR | min^-1^ | 12-16 | 12 | 22 |
| TV | ml | 400-700 | 430 | 800 |
| etCO2 | kPa | 4.2-5.5 | 5.1 | 3.3 |
| T | °C | 36.5-37.4 | 34.9 | 36.8 |

**Online Resource 3** 2 Scenarios used in the simulation sessions

*ST Segment Elevation equals 4.5 mV in leads II, III, aVF

HR: heart rate; ST: ST segment; MAP: mean arterial pressure; CVP: central venous pressure; BIS: bispectral index; TOF: train-of-four ratio; SpO2: oxygen saturation; RR: respiratory rate; TV: tidal volume; etCO2: expiratory carbon dioxide concentration; T: body temperature; min^-1^: per minute; mV: millivolt; mmHg: millimeter mercury; ml: milliliter; kPa: kilopascal; °C: degree Celsius


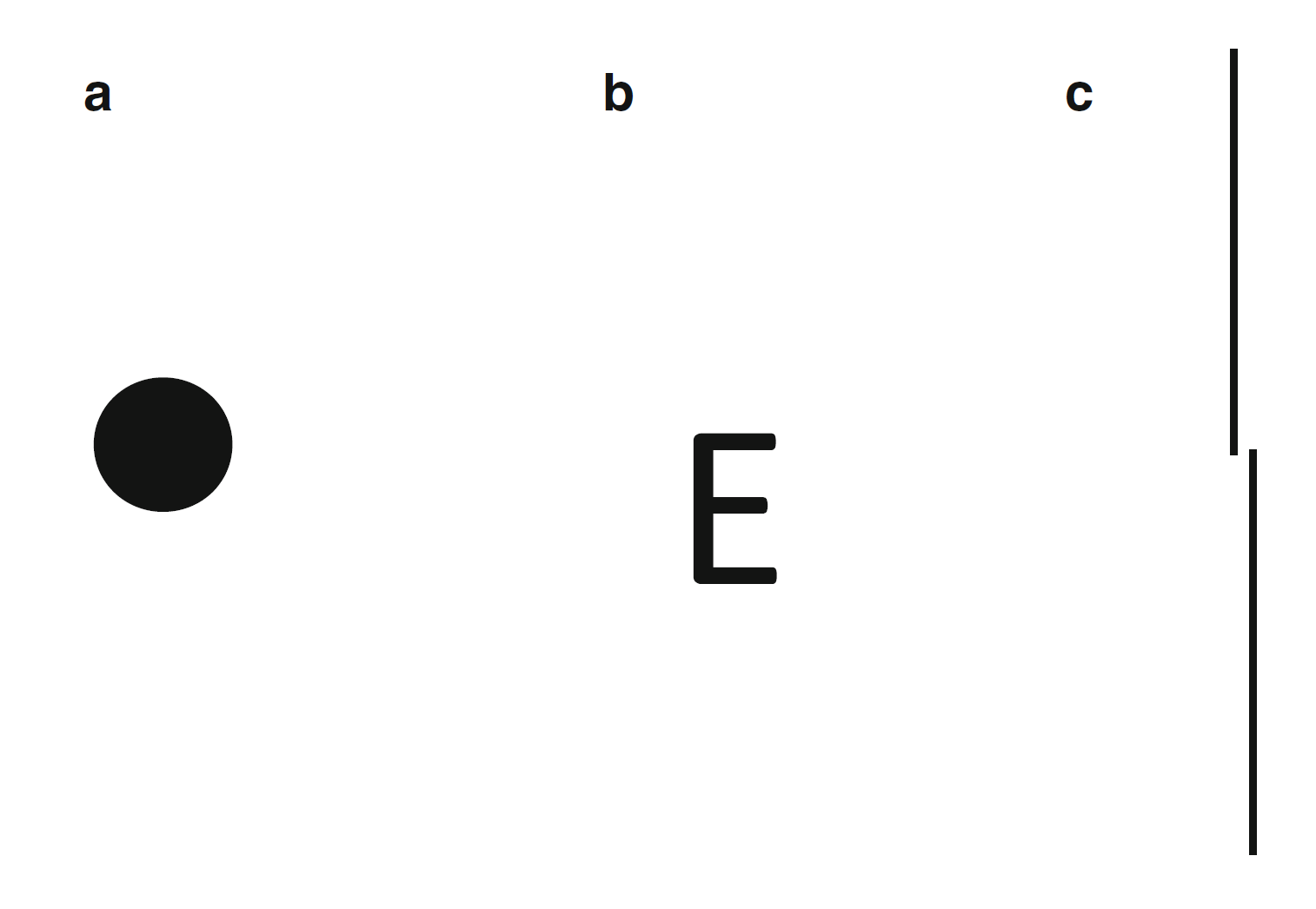


**Online Resource 4** Schematic representation of different types of visual acuity: minimal visible – is there a dot? (a); minimal resolvable – is that an E or an F? (b); minimal discriminable visual acuity – is the upper line to the left or right of the lower line? (c). Adapted from [Skalicky, S. E.]. (2016). Ocular and Visual Physiology (p. 275). Springer Singapore

| Heart rate | Minimum visible* |
| --- | --- |
| ST segment | Minimum visible |
| Mean arterial pressure | Minimum discriminable |
| Central venous pressure | Minimum discriminable |
| Bispectral index | Minimum visible |
| Train-of-four ratio | Minimum resolvable |
| Oxygen saturation | Minimum visible |
| Respiratory rate | Minimum visible* |
| Tidal Volume | Minimum discriminable |
| Expiratory carbon dioxide concentration | Minimum discriminable |
| Body temperature | Minimum visible |

**Online Resource 5** Pairing of the Philips Visual Patient Avatar vital sign visualizations with their appropriate type of visual acuity. We marked the pulse and respiratory rates as frequency-dependent vital signs (*)
